# Strong Regionality and Dominance of Anaerobic Bacterial Taxa Characterize Diazotrophic Bacterial Communities of the Arcto-Alpine Plant Species *Oxyria digyna* and *Saxifraga oppositifolia*

**DOI:** 10.3389/fmicb.2017.01972

**Published:** 2017-10-13

**Authors:** Manoj Kumar, Jan Dirk van Elsas, Riitta Nissinen

**Affiliations:** ^1^Department of Biological and Environmental Science, University of Jyväskylä, Jyväskylä, Finland; ^2^Department of Microbial Ecology, University of Groningen, Groningen, Netherlands

**Keywords:** *nifH*, pioneer plants, *Geobacter*, *Clostridium*, endophytic bacteria

## Abstract

Arctic and alpine biomes are most often strongly nitrogen-limited, and hence biological nitrogen fixation is a strong driver of these ecosystems. Both biomes are characterized by low temperatures and short growing seasons, but they differ in seasonality of solar radiation and in soil water balance due to underlying permafrost in the Arctic. Arcto-alpine plant species are well-adapted to the low temperatures that prevail in their habitats, and plant growth is mainly limited by the availability of nutrients, in particular nitrogen, due to slow mineralization. Nitrogen fixing bacteria are likely important for plant growth in these habitats, but very little is known of these bacteria or forces shaping their communities. In this study, we characterized the potential nitrogen fixing bacterial (PNFB) communities associated with two arcto-alpine pioneer plant species, *Oxyria digyna* (mountain sorrel) and *Saxifraga oppositifolia* (blue saxifrage), in three climate regions. Both of these plants readily colonize low nutrient mineral soils. Our goal was to investigate how climate (region) and, on the other hand, host plant and plant species shape these communities. To our knowledge, this is the first comprehensive study describing PNFB communities associated with pioneer plants in different arcto-alpine biomes. Replicate samples were taken from two arctic regions, Kilpisjärvi and Ny-Ålesund, and one alpine region, Mayrhofen. In these, the PNFB communities in the bulk and rhizosphere soils and the plant endospheres were characterized by *nifH*-targeted PCR and massive parallel sequencing. The data revealed strong effects of climatic region on the dominating nitrogen fixers. Specifically, *nifH* sequences related to *Geobacter* (δ-*Proteobacteria*) were present in high relative abundances in the nitrogen-fixing communities in the Mayrhofen and Kilpisjärvi regions, while members of the *Clostridiales* prevailed in the Kilpisjärvi and Ny-Ålesund regions. The bulk and rhizosphere soil as well as the endosphere communities in the Mayrhofen region were all characterized by high relative abundances of *nifH* sequences related to *Geobacter*. In contrast, the endosphere and soil (bulk or rhizosphere soil) communities in the High Arctic were highly divergent: endosphere communities in the arctic regions were shaped by *Clostridium* spp., while *nifH* sequences representing δ-*Proteobacteria*, β-*Proteobacteria, Cyanobacteria* (in Ny-Ålesund), and *Verrucomicrobia* (in Kilpisjärvi) dominated the soil communities. Interestingly, the major PNFB genera identified in this study have been previously identified as members of conserved core microbiomes in the endospheres and seeds of these plants by 16S rRNA gene based analyses combined with bacterial isolation, suggesting a very tight interaction between diazotrophic bacteria and these arctic pioneer plants. Overall, anaerobic bacterial taxa dominated the PNFB communities of the endospheres and rhizospheres of the two plant species in all study sites. This could indicate anoxic conditions in and around plant roots at the time of sampling (early growth season), created by melting snow and underlying permafrost.

## Introduction

Nitrogen (N) is considered to be the major limiting factor for both microbial and plant growth in arctic and alpine biomes (Chapin et al., 1986; Wallenstein et al., 2009; Sistla et al., 2012). In particular, the amount of available soil nitrogen is very low in low organic content mineral-rich tundra soils. One example is given by recently deglaciated soils with low levels of (mineralizable) nitrogen and carbon that are colonized by patchy pioneer plant communities (Brankatschk et al., 2011). In these soils, nitrogen fixation is the primary source of bioavailable nitrogen (N) for plants (Chapin et al., 1992). Furthermore, the levels of nitrogen fixation and N cycling were shown to be correlated with vegetation cover by Brankatschk et al. (2011). These authors observed no N-fixation activity, and extremely low denitrification and nitrification rates, coupled to low *nifH* gene copy numbers, in the youngest (10-year old) soils in glacier forefields. In contrast, a densely plant-covered 120-year old soil in the same chronosequence showed both higher enzyme activities and higher copy numbers of the *nifH*, next to *nirK* and *nirS*, genes. Clearly, the older soils had gained the capacity to cycle nitrogen on the basis of a pioneer-species-based ecosystem build-up, in which plants found capacities to grow and, in turn, supported the evolved microbial communities and activity.

Arctic plants are well-adapted to low temperatures during the growing season. In fact, the photosynthesis rates in the Arctic are comparable to those at lower latitudes (Central Europe) (Chapin et al., 1987). However, to compensate for the slower enzyme kinetics at low temperatures, higher amounts of enzymes are required. For example, arctic plants have significantly higher numbers of the key photosynthesis enzyme RuBisCO (ribulose-1, 5 bisphosphate-carboxylase-oxygenase). Consequently, the tissues of arctic plants contain more N than those of plants in warmer climates (Weintraub and Schimel, 2005), underlining the demand for N availability. Biological nitrogen fixation thus constitutes a key strategy involved in initial primary production. In the emerged systems, with plants in place as primary producers, the key nitrogen input will mainly come from plant-symbiotic and/or associative nitrogen fixers. The fixed nitrogen may then be recycled/mineralized via deamination (yielding ammonia), followed by nitrification and denitrification reactions. Associative nitrogen fixing bacteria are likely vital for plant growth in the arcto-alpine pioneer communities. Yet, very little is known about these bacteria, their diversity, or forces shaping their communities.

In previous studies, we focused on two arcto-alpine pioneer plant species, *Oxyria digyna* and *Saxifraga oppositifolia*, as “models” of the primary colonizers of the nutrient-poor mineral soils that abound in arctic and alpine regions. Preliminary evidence was found for the contention that the soil bacterial communities in different arcto-alpine regions are region-specific, whereas endophytic bacterial communities (using the 16S rRNA gene sequence as a proxy) are host plant specific (Kumar et al., 2017). However, these communities also share a group of bacterial taxa (core microbiome), which are apparently associated with the host plants in all three climatic regions, including several potential nitrogen fixers (Kumar et al., 2017).

In this study, we examined the factors shaping the communities of potential nitrogen fixing bacteria (PNFB) in the two selected arcto-alpine pioneer plants and the associated soils in different climate zones. We hypothesized that the bacterial taxa with known nitrogen fixers, which we had previously identified as major members of the core microbiomes of these pioneer plants are also major part of the *nifH* communities of these plants. Further, we hypothesized, that, like the total bacterial communities, the PNFB communities are host plant specific, and additionally shaped by climatic regions.

## Materials and methods

### Sampling and DNA isolation

**Figure 1:**
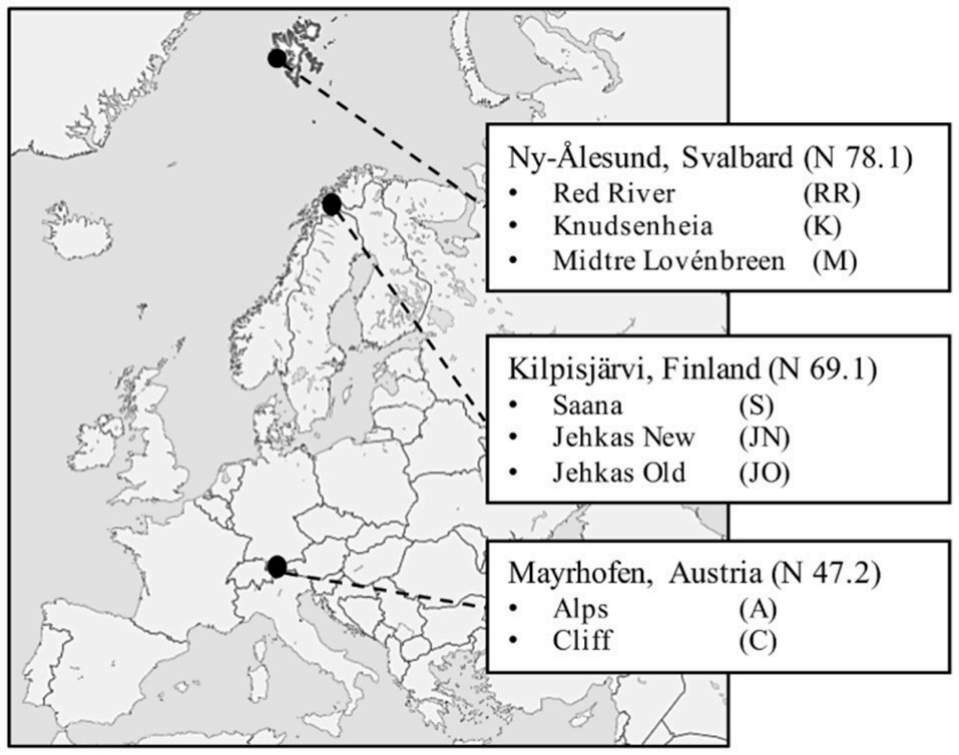
Sampling locations: Mayrhofen in Austrian Alps, Kilpisjärvi in low-arctic Finnish Lapland and Ny-Ålesund in high-arctic Svalbard.

The plant and DNA samples used to characterize total bacterial communities (Kumar et al., 2017) were also used in this study, and details of sampling sites can be found in the supplemental data in Kumar et al. (2017). Samples were collected using random sampling strategy in June 2012 from eight sampling sites in three different geographic regions: Kilpisjärvi, Northern Finland (KJ), Ny-Ålesund, Svalbard (NÅ), and Mayrhofen, Austria (MA) (Figure 1). NÅ is located in the high Arctic climatic region, where the mean annual temperature is −6.4 and soil temperatures do not rise above 10°C. The vegetation in NÅ region is arctic tundra, and plant growing season typically 60–90 days. Sampling sites in KJ represent the low Arctic climate region. The mean annual temperatures in KJ is −2.1°C, with high seasonal temperature fluctuations. Growing season in the tundra vegetation zone in KJ is up to 90 days. The MA sites represent alpine climate, and are located in the alpine tundra vegetation zone, above the tree-line in the Austrian Alps, at an elevation of ca. 2,400 m. Coordinates of the sampling sites can be found at: https://www.frontiersin.org/articles/10.3389/fmicb.2017.00012/full#supplementary-material.

Six replicates of bulk soil and plant samples of each *O. digyna* and *S. oppositifolia* (with adhering rhizosphere soil) were collected from all sites and transported to the laboratory. All samples were processed as described in Kumar et al. (2017). In brief, after removing rhizosphere soil parts, plant roots were thoroughly washed with water and surface-sterilized by immersion into 3% sodium hypochlorite (3 min), followed by rinses in sterile double-distilled water (3 × 90 s). 80–100 mg of processed root sample were snap-frozen with liquid nitrogen and stored at −80°C for DNA extraction and analysis.

### nifH library preparation and sequencing

The MoBio Power soil kit (MoBio, Carlsbad, CA, USA) and Invisorb Spin plant Midi kit (STRATEC, Biomedical AG, Germany) were used to extract DNA from soil and plant samples, respectively, as described in Kumar et al. (2017). Libraries for sequencing were prepared using the M-13 linker method by Mäki et al. (2016). The *nifH* gene was amplified using an optimized nested approach. The first PCR was done with primers 19f/nifH3 (Yeager et al., 2004) and the second one with one forward primer nifH1f (as described by Zehr and McReynolds, 1989) with M13 linker and equimolar concentration of two reverse primers, nifH2r (Zehr and McReynolds, 1989) and nifH2 (Izquierdo and Nüsslein, 2006). The first PCR reaction had 2 μl of template DNA, 1x PCR buffer, 2.5 mM of MgCl2, 1 mg/ml of bovine serum albumin (BSA), 0.4 mM dNTP's, 0.9 μM of each primer, and 2,500 U/ml of GoTaq DNA ploymerase (Promega, WI, USA) in a 30 μl reaction volume. For the second PCR, 1 μl of amplified product from the first PCR was added along with 1x PCR buffer, 1 mM of MgCl2, 1 mg/ml of BSA, 0.2 mM dNTP's, 0.9 μM of each primer and 1250 U/ml of GoTaq DNA ploymerase (Promega) in a 30 μl reaction volume. Amplifications for both PCR reactions were performed as follows: 2 min denaturation at 95°C followed by 35 cycles of denaturing, annealing and extension at 95°C for 1 min, 51°C (first PCR)/53°C (second PCR) for 1 min and 72°C for 1 min, respectively. Final extension was carried out at 72°C for 2 min.

Samples were barcoded using a third PCR round, where an aliquot (1 μl) of the second PCR was re-amplified using the barcode-M13 as forward primer and nifH2r/nifH2-P1 with adaptor A as reverse primer. PCR mix and conditions were similar to those described for the second PCR, except that only eight cycles were used for amplification. The products were purified using the Agencourt AMPure XP PCR purification system (Beckman Coulter, CA, USA), followed by quantification with a Qubit Fluorometer (Invitrogen). Equivalent DNA quantities of each sample were then pooled and size-fractionated (size selection range of 350–550 bp) using Pippin Prep (Sage Science, MA, USA) with 2% agarose gel cassette (Marker E) according to the manufacturer's protocol. The libraries were sequenced using an Ion 314 chip kit V2 BC on an Ion Torrent PGM machine (Life Technologies, MA, USA) in the Biocenter, Oulu, Finland. Nucleotide sequence data has been submitted to the ENA database with accession number PRJEB20618.

### Bioinformatics and statistical analysis

All reads from the Ion Torrent sequencing were processed using QIIME (Quantitative Insights Into Microbial Ecology, Caporaso et al., 2010) and Fungene pipelines (Fish et al., 2013), as described by Zhang et al. (2015). All sequences with quality scores below 22 and lengths below 240 bp were removed. USEARCH (Edgar, 2010) algorithm was used for chimera removal. After chimera removal, all nucleotide sequences were translated into protein sequences. Frameshifts were detected and corrected with Framebot (length cutoff = 80 AAs), aligned using HMMER3 Aligner (with nifH as representative gene) and then clustered by RDP mcClust (90% similarity) in the Fungene pipeline at RDP (fungene.cme.msu.edu). The resulting cluster file was converted into an OTU table by a RDP cluster file formatter in R (version 3.2.5; https://www.r-project.org/). OTUs were assigned to the nearest neighbor by FrameBot in Fungene pipeline. The OTUs were grouped into OPUs (operating phylogenetic units) based on clustering with the same reference sequence. Taxonomic affiliations of 12 OPUs were complemented by P-BLAST at NCBI (blast.ncbi.nlm.nih.gov).

Prior to community analysis, the sample reads werestandardized by subsampling all the samples with read counts above dataset median to the median. The samples with reads below median were included as such, as described in Cárcer et al. (2011). OPU abundances were square-root transformed prior to community analyses. ANOVA (analysis of variance), and PERMANOVA (permutational multivariate analysis of variance) and PCoA (Principal Co-Ordinate Analysis) were used to test and visualize the differences between the sample groups. The impact of OPUs to similarities within and dissimilarities between sample groups was estimated with SIMPER (Similarity Percentages–species contributions). Community analyses were all performed in the Primer 6.1. software package (http://www.primer-e.com/).

## Results

Regions (350 bp long) of the *nifH* genes were amplified from microbial community DNA isolated from the bulk and rhizosphere soils and endospheres from *O. digyna* and *S. oppositifolia* in the three regions (Figure 1) using the optimized PCR approach (described in Materials and Methods). The quality-trimmed and translated *nifH* sequences were used for clustering, resulting in a total of 296 OTUs (at 90% amino acid sequence identity). These 296 OTUs were further clustered based on alignments with NifH protein reference sequences in the RDP FunGene NifH database. This resulted in 97 units, denoted “operational phylogenetic units” (OPUs).

**Figure 2:**
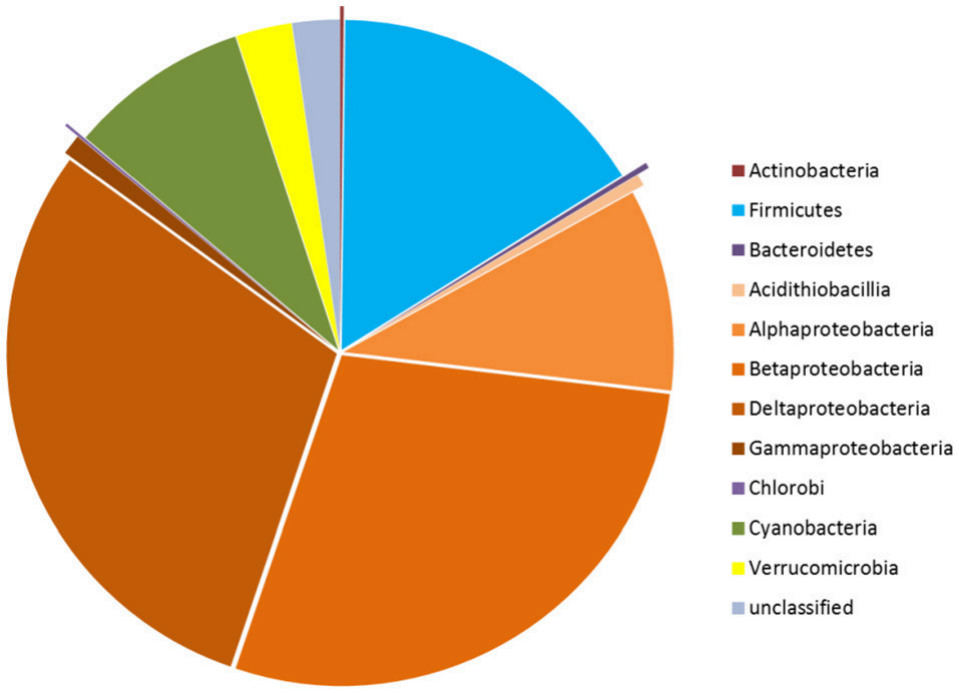
Taxonomic distribution of the *nifH* gene sequences in the three geographic regions at phylum level, except for phylum Proteobacteria which is presented at class level.

Considering all the OPU data, a high diversity of *nifH* gene sequences was detected in our soil and plant microbial communities. Thus, a suite of *nifH* genes, affiliated with the bacterial phyla *Proteobacteria (classes* α*-*, β*-*, δ-, and γ*-Proteobacteria* and *Acidithiobacillia), Firmicutes, Actinobacteria, Chlorobi, Bacteroidetes, Cyanobacteria*, and *Verrucomicrobia*, was detected (Figure 2). The most abundant reads in the dataset clustered with *nifH* gene sequences from the δ-*Proteobacteria* (genera *Geobacter* and *Desulfotignum*), γ*-Proteobacteria* (*Bradyrhizobium*), *Firmicutes* (genera *Clostridium, Desulfosporosinus* and *Acetobacterium*) and β*-Proteobacteria* (*Burkholderia* [*Burkholderiales*] and *Leptothrix* [*Comamonadaceae*]) (Figure 2). In the further text, we use these partial sequences to characterize the populations of “PNFB.”

### The species richness and diversity of PNFB communities is lowest in the high arctic

**Figure 3:**
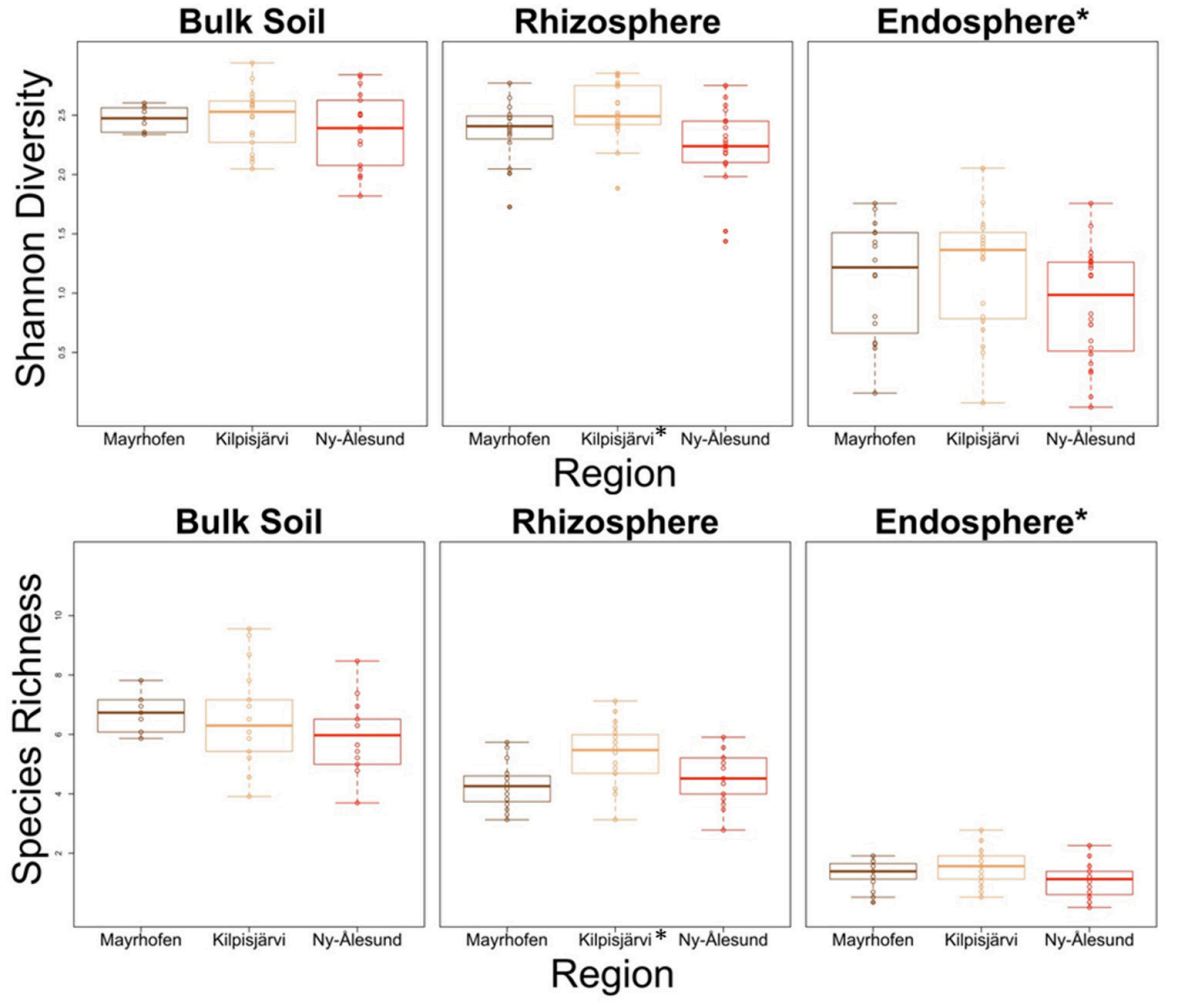
Species richness and Shannon diversity of PNFB communities in different regions and compartments. Diversity indices were calculated using *nifH* gene OPUs as a proxy. Compartment or regions with differ significantly from other groups are marked by asterisk.

The species richness (SR) and Shannon diversity values (SD) of the PNFB communities (based on the *nifH* gene amplicon OPUs as a proxy) were highest in the bulk soils, followed by the rhizosphere soils and the endosphere samples. Specifically, the SR and SD values in the endosphere were significantly lower than those in the two soil compartments (*P* < 0.05; Figure 3). Then, the richness and diversity values of the communities from the three climate regions were compared separately for each compartment. The SR and SD values of the bulk soil communities were both highest in the MA, but the differences were not significant. In the plant-associated compartments (rhizo- and endospheres), the values of both parameters were highest in the KJ in both plant-associated compartments, but the differences between KJ and other regions were significant only in the rhizosphere communities (*P* < 0.05; Figure 3).

### Community structures of PNFB are driven by compartment and region

**Figure 4:**
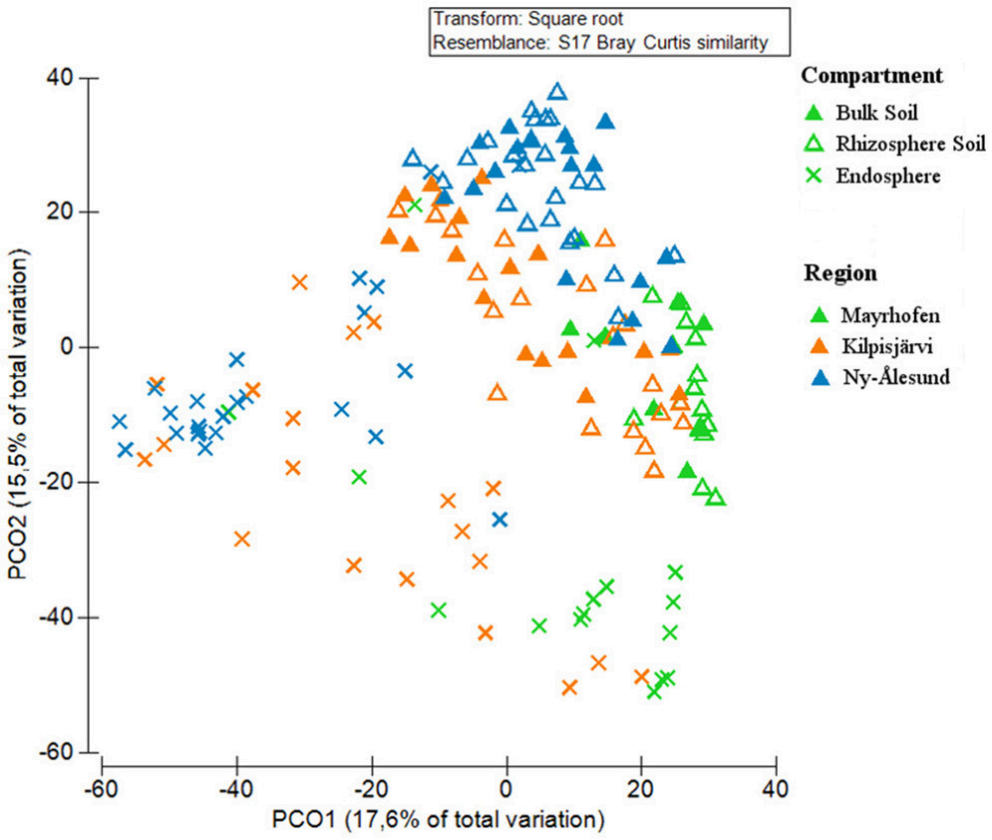
Principal Coordinate Analysis (PCoA) of PNFB communities, from bulk soils, rhizosphere soils, and endospheres of *Oxyria digyna* and *Saxifraga oppositifolia* from three regions, based on Bray-Curtis similarity matrix of square root transformed relative abundances of *nifH-*based OPUs. Symbol shapes correspond to different compartments, and symbol colors to different regions.

The community structures of the PNFB were analyzed at the OPU level across all samples. Compartment, region and sampling site all had significant impacts on the PNFB community structures, with compartment being the most important factor (Table 1). The endosphere PNFB communities were significantly different from those from both soil compartments (*P* = 0.003), while we detected no significant differences between bulk and rhizosphere soil community structures (Table 1) in region-wide analyses. This was also evident in the PCoA ordination, which showed that the endosphere communities grouped separately from the soil-derived ones (Figure 4).

**Table 1:** Impact of different factors on PNFB community structures.

**PERMANOVA**			
**GLOBAL ANALYSIS (ALL SAMPLES)**			
**Factor**	***F***	***P***	**q S**
Co	8.5428	0.001	23.367
Re	3.63731	0.01	20.308
Si(re)	4.3384	0.001	17.751
co × re	2.0550	0.002	14.932
co × si(re)	1.9712	0.001	16.432
**PAIR-WISE ANALYSES**			
**Compartments**	**Bulk vs. Rhizo t (P)**	**Bulk vs. Endo t (P)**	**Rhizo vs. Endo t (P)**
All regions	NS (0.189)	3.0884 (0.003)	3.3799 (0.003)
Mayrhofen	1.4788 (0.019)	2.2824 (0.001)	2.7259 (0.001)
Kilpisjärvi	1.6801 (0.014)	3.2609 (0.001)	2.9407 (0.001)
Ny-Ålesund	1.5336 (0.018)	3.1962 (0.001)	3.6902 (0.001)
**Regions**	**Mayrhofenx Kilpisjärvi t (P)**	**Mayrhofenx Ny-Ålesund t (P)**	**Kilpisjärvix Ny-Ålesund t (P)**
All compartments	3.0703 (0.001)	4.000 (0.001)	3.0815 (0.001)
Bulk soil	2.3047 (0.002)	2.155 (0.001)	2.8326 (0.001)
Rhizosphere	2.9113 (0.001)	3.8324 (0.001)	3.1553 (0.001)
Endosphere	2.1865 (0.001)	2.9723 (0.001)	1.9935 (0.001)
**RHIZOSPHERE AND ENDOSPHERE**			
**Factor**	***F***	***P***	**sqS**
Co	21.769	0.001	27.875
Pl	5.0168	0.001	12.259
Re	12.096	0.001	24.787
co × pl	3.2908	0.001	13.092
co × re	3.9875	0.001	18.189
pl × re	1.7684	0.015	9.2244
co × pl × re	1.9635	0.009	14.608

*The PERMANOVA is based on Bray-Curtis dissimilarity matrixes of square-root transformed relative abundances of PNFB OPUs. Co, compartment; re, region; si, site; pl, plant species. Nested factors are denoted in brackets () and test for interaction between factors is denoted with x*.

In addition to compartment, a clear trend for grouping according to region was also visible in the PCoA, with NÅ and MA samples clustering at opposite sites on the first PCO (Figure 4). The communities in the three climatic regions differed significantly from each other in both global analysis and when different compartments were analyzed separately (Table 1). The plant-associated KJ communities were more similar to both the NÅ and MA communities than the latter two to each other, while the opposite was true for the bulk soil communities (Table 1).

### Different bacteria dominate the PNFB communities in different regions and compartments

**Figure 5:**
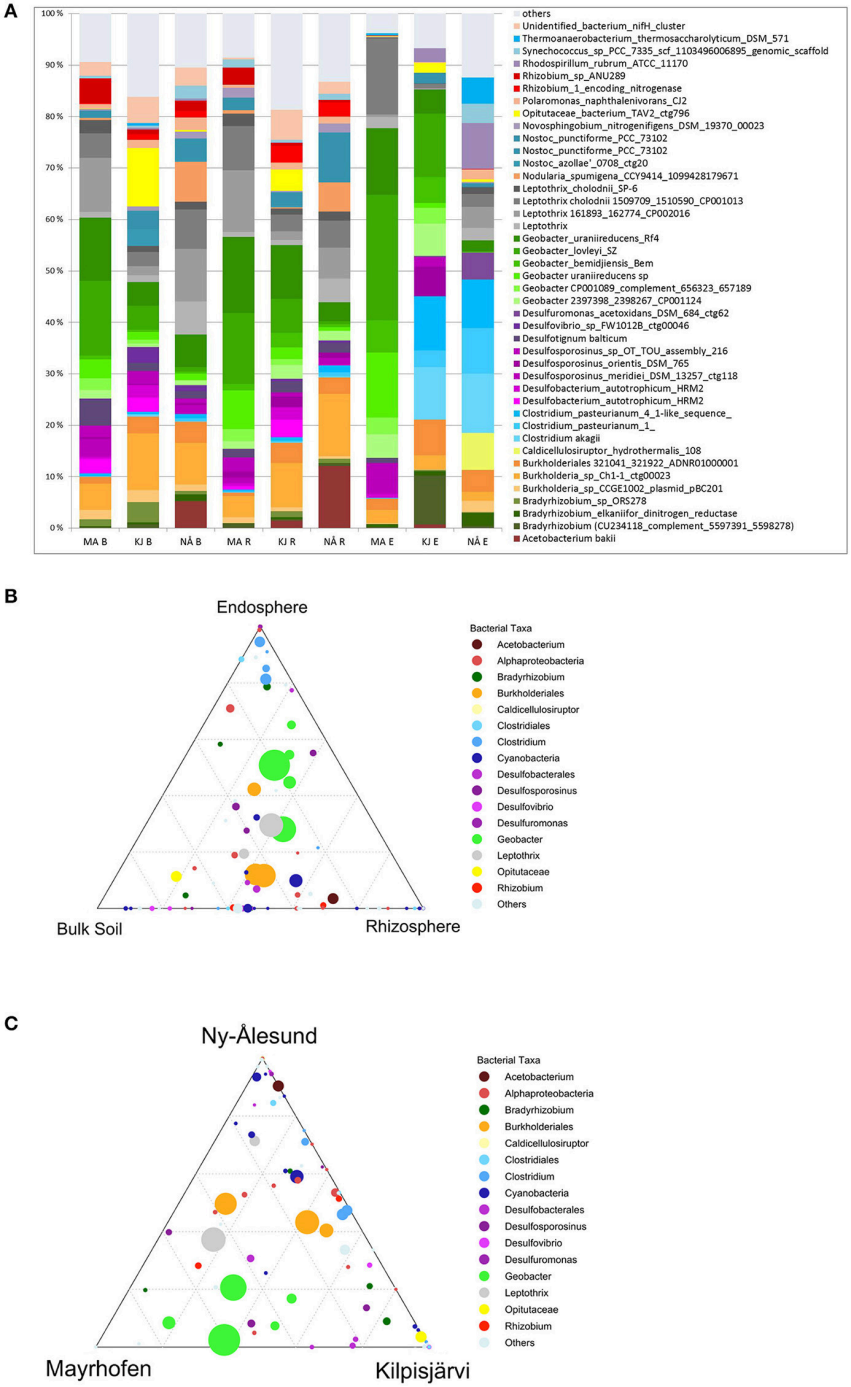
Average relative abundances **(A)** and distribution of OPUs **(B,C)** in different sample compartments and geographic regions. **(A)** Average relative abundances (four biological replicates) of PNFB communities in different sample compartments and geographic regions. Compartments: B, bulk soil; R, rhizosphere soil; E, endosphere; MA, Mayrhofen; the Alps; KJ, Kilpisjärvi; Low Arctic, NÅ, Ny-Ålesund; High Arctic. Only major PNFB OPUs (relative abundance above 1%) are indicated, OPUs with <1% relative abundance are grouped together (“others”). **(B)** Ternary plot of distribution of OPUs across different compartments. Each circle represents one OPU. The size of the circle represents the average relative abundance of the OPU in the dataset. The circle color corresponds to OPUs taxonomic affiliation. Location of the circle in the plot in relation to different compartments indicates the contribution of each compartment to the OPU's total relative abundance, with the dotted grid inside the plot area indicating 20% increments of contribution. **(C)** Ternary plot of distribution of OPUs across different regions. Similar to **(B)**, the size, the color and the location of each circle indicates the average relative abundance, taxonomic affiliation and association with different regions of the corresponding OPU.

We next investigated the taxonomic composition of the PNFB communities across the different compartments and regions. The bulk and rhizosphere soil PNFB communities were characterized by high relative abundances of *nifH* sequences representing the Betaproteobacterial taxa *Leptothrix* and *Burkholderia*, as well as the Deltaproteobacterial genus *Geobacter* in all three regions (Figure 5). Several taxa, however, were highly unevenly distributed between the different regions. *Geobacter*-type *nifH* sequences dominated the bulk and rhizosphere soils in MA and were also abundant in KJ soils, but they were detected only at very low relative abundances in NÅ (Figure 5, Table 2). In contrast, *Acetobacterium bakii* (*Clostridiales*), and *Cyanobacteria*-related *nifH* sequences (*Nostoc, Nodularia, Synechococcus*) were more abundant in the NÅ region than in the other regions (Figure 5C). Furthermore, OPUs clustering with *nifH* sequences from the *Opitutaceae* (*Verrucomicrobia*) were highly abundant in the KJ soils (Figure 5). These taxa also defined the region-specific soil communities (Table 2).

**Table 2:** Top OPUs contributing up to 90% (endosphere) or 60% (rhizosphere and endosphere) cumulative similarity of PNFB communities in different regions are shown.

**Species**	**Av.Ab**	**Av.Sim**	**Sim/SD**	**Contrib (%)**	**Cum. (%)**
**BULK SOIL**					
**Group Mayrhofen**					
*Geobacter_lovleyi*_SZ	3.73	6.44	2.09	12.32	12.32
*Geobacter_uraniireducens*_Rf4	3.26	5.47	1.74	10.48	22.80
*Burkholderia*_sp_CCGE1002_plasmid_pBC201	3.07	4.66	1.47	8.91	31.71
*Burkholderia*_sp_Ch1-1_ctg00023	2.45	4.17	2.43	7.98	39.69
*Leptothrix_cholodnii*_SP-6	1.56	3.19	9.31	6.11	45.80
*Desulfotignum balticum*	2.02	2.92	1.52	5.60	51.40
*Rhizobium*_sp_ANU289	1.88	2.85	1.35	5.46	56.85
*Geobacter uraniireducens* sp	1.66	2.50	1.52	4.79	61.64
**Group Kilpisjärvi**					
*Burkholderia*_sp_Ch1-1_ctg00023	3.58	6.75	2.44	16.64	16.64
*Opitutaceae*_bacterium_TAV2_ctg796	3.55	4.90	1.15	12.57	29.21
*Bradyrhizobium*_sp_ORS278	1.70	2.59	1.27	6.37	35.58
*Burkholderia*_sp_CCGE1002_plasmid_pBC201	1.68	2.48	1.57	6.12	41.70
*Geobacter_uraniireducens*_Rf4	1.74	2.03	1.06	5.00	46.70
*Leptothrix*	1.02	1.70	1.52	4.20	50.90
Unidentified_bacterium_nif_cluster	1.46	1.35	0.65	3.33	54.23
*Desulfovibrio*_sp_FW1012B_ctg00046	1.28	1.34	0.65	3.31	57.54
*Geobacter_lovleyi*_SZ	1.48	1.17	0.47	2.88	60.42
**Group Ny-Ålesund**					
*Burkholderia*_sp_Ch1-1_ctg00023	3.39	6.56	2.12	15.30	15.30
*Burkholderia*_sp_CCGE1002_plasmid_pBC201	3.19	6.15	2.32	14.33	29.63
*Leptothrix*	2.36	4.25	1.92	9.92	39.55
*Synechococcus*_sp_PCC_7335	1.41	2.23	1.41	5.19	44.74
*Leptothrix*_*cholodnii*_SP-6	1.12	1.86	1.49	4.32	49.06
*Nostoc_punctiforme*_PCC_73102	1.59	1.71	0.75	3.98	53.04
*Acetobacterium bakii*	1.74	1.65	0.60	3.84	56.87
*Polaromonas_naphthalenivorans*_CJ2	1.25	1.62	1.03	3.78	60.65
**RHIZOSPHERE SOIL**					
**Group Mayrhofen**					
*Geobacter_uraniireducens*_Rf4	3.80	8.09	2.86	14.08	14.08
*Geobacter_lovleyi*_SZ	3.88	7.67	2.30	13.36	27.44
*Burkholderia*_sp_CCGE1002_plasmid_pBC201	3.51	7.06	1.99	12.30	39.73
*Geobacter uraniireducens* sp	2.73	5.80	3.07	10.09	49.82
*Burkholderia*_sp_Ch1-1_ctg00023	2.11	3.96	2.79	6.89	56.71
*Rhizobium*_sp_ANU289	1.66	2.67	1.36	4.65	61.37
**Group Kilpisjärvi**					
*Burkholderia*_sp_Ch1-1_ctg00023	3.30	6.00	2.53	14.96	14.96
*Geobacter_bemidjiensis*_Bem	2.17	3.55	1.42	8.86	23.82
*Geobacter_uraniireducens*_Rf4	2.58	3.40	1.17	8.50	32.32
*Geobacter_lovleyi*_SZ	2.33	2.94	0.91	7.34	39.66
*Burkholderia*_sp_CCGE1002_plasmid_pBC201	1.55	2.67	2.32	6.67	46.34
*Desulfobacterium_autotrophicum*_HRM2	1.90	2.64	1.08	6.58	52.91
*Opitutaceae*_bacterium_TAV2_ctg796	1.41	1.23	0.50	3.06	55.98
*Bradyrhizobium*_sp_ORS278	0.89	1.18	1.03	2.94	58.91
*Leptothrix_cholodnii*_SP-6	0.95	1.15	0.99	2.87	61.79
**Group Ny-Ålesund**					
*Burkholderia*_sp_Ch1-1_ctg00023	3.77	7.83	2.68	17.78	17.78
*Acetobacterium bakii*	2.94	4.36	0.95	9.90	27.68
*Burkholderia*_sp_CCGE1002_plasmid_pBC201	2.22	3.60	1.39	8.18	35.86
*Nostoc_punctiforme*_PCC_73102	2.50	3.51	1.05	7.97	43.83
*Leptothrix*	1.92	3.16	1.59	7.17	50.99
*Nodularia_spumigena*_	1.80	2.02	0.67	4.59	55.58
*Leptothrix_cholodnii*_SP-6	1.18	1.91	1.51	4.35	59.93
*Synechococcus*_sp_	0.95	1.61	1.61	3.65	63.58
**ENDOSPHERE**					
**Group Mayrhofen**					
*Geobacter_lovleyi*_SZ	4.17	10.91	1.01	35.37	35.37
*Geobacter_uraniireducens*_Rf4	2.96	7.16	0.96	23.21	58.59
*Geobacter uraniireducens* sp	2.62	5.85	0.88	18.98	77.56
*Geobacter_bemidjiensis*_Bem	2.27	3.15	0.45	10.22	87.78
*Bradyrhizobium_elkaniifor*_	1.41	1.14	0.27	3.71	91.49
**Group Kilpisjärvi**					
*Geobacter_bemidjiensis*_Bem	2.52	4.21	0.60	18.17	18.17
*Clostridium_pasteurianum*_4_1-like_sequence_	2.23	3.55	0.63	15.31	33.48
*Geobacter_lovleyi*_SZ	2.21	3.04	0.51	13.10	46.58
*Bradyrhizobium*	2.27	2.89	0.34	12.46	59.05
*Clostridium akagii*	1.73	2.50	0.53	10.78	69.83
*Clostridium_pasteurianum*_1_	1.14	1.83	0.52	7.91	77.74
*Bradyrhizobium_elkaniifor*	0.67	0.90	0.39	3.88	81.62
*Geobacter_uraniireducens*_Rf4	0.98	0.87	0.33	3.74	85.36
*Burkholderia*_sp_Ch1-1_ctg00023	1.23	0.72	0.22	3.12	88.48
*Desulfosporosinus_orientis*_DSM_765	0.86	0.53	0.27	2.29	90.77
**Group Ny-Ålesund**					
*Clostridium_pasteurianum*_4_1-like_sequence_	2.48	4.72	0.58	26.10	26.10
*Bradyrhizobium_elkaniifor*	2.41	4.25	0.49	23.48	49.58
*Clostridium akagii*	2.31	3.60	0.47	19.87	69.45
*Opitutaceae*_bacterium_TAV2_ctg796	0.88	1.49	0.47	8.21	77.66
*Clostridium_pasteurianum*_1_	1.21	0.91	0.23	5.01	82.67
*Nostoc_punctiforme*_PCC_73102	0.44	0.53	0.35	2.92	85.59
*Burkholderia*_sp_Ch1-1_ctg00023	0.82	0.39	0.18	2.16	87.75
*Burkholderia*_sp_CCGE1002_plasmid_pBC201	0.76	0.30	0.14	1.66	89.40
*Polaromonas_naphthalenivorans*_CJ2	0.49	0.28	0.22	1.55	90.95

*The data are based on square root transformed relative abundances of OPUs*.

The endosphere PNFB communities were dominated by *nifH* OPUs representing the genera *Burkholderia, Bradyrhizobium, Geobacter* (*Deltaproteobacteria*), and *Clostridium* (*Firmicutes*) (Figure 5A). *Clostridium* OPUs were mostly restricted to the endosphere, while *Burkholderia* and *Geobacter* were present in all comparments (Figure 5B). However, the latter two taxa were highly unevenly distributed across the climatic regions. While the MA communities were strongly dominated by OPUs related to several *Geobacter* species, including *G. lovleyi, G. bemidjiensis*, and *G. uraniireducens*, the NÅ endosphere communities were strongly shaped by the genus *Clostridium* (*3 OPUs*; Figure 5A, Table 2). Such *Clostridium*-like OPUs were virtually absent from the MA endosphere communities, and, likewise, *Geobacter*-type OPUs in NÅ (Figure 5C). Moreover, the major *nifH* OPUs in the KJ endosphere communities were affiliated with both the genera *Clostridium* and *Geobacter*, next to *Bradyrhizobium*, and *Burkholderia* (Figure 5, Table 2).

The endosphere and soil communities were more dissimilar from each other in NÅ (pseudo-F = 9.79, *P* = 0.001) than in the other regions (pseudo-F = 7.80 *P* = 0.001 and 5.56 *P* = 0.001 for KJ and MA, respectively). While in the MA and—to a lesser extent—in the KJ microbiomes, the major PNFB OPUs (representing *Geobacter, Burholderiales*, and *Leptothrix*) were found in all compartments, the OPUs dominating the endosphere in NÅ (*Clostridum*) were barely detectable in the corresponding soil communities. Moreover, several of the major soil OPUs (e.g., *Acetobacter*) were virtually absent from the endosphere (Figure 5B, Table 2).

### *Geobacter* and *Bradyrhizobium* diazotrophs are differentially enriched in the *S. oppositifolia* and *O. digyna* endospheres

**Figure 6:**
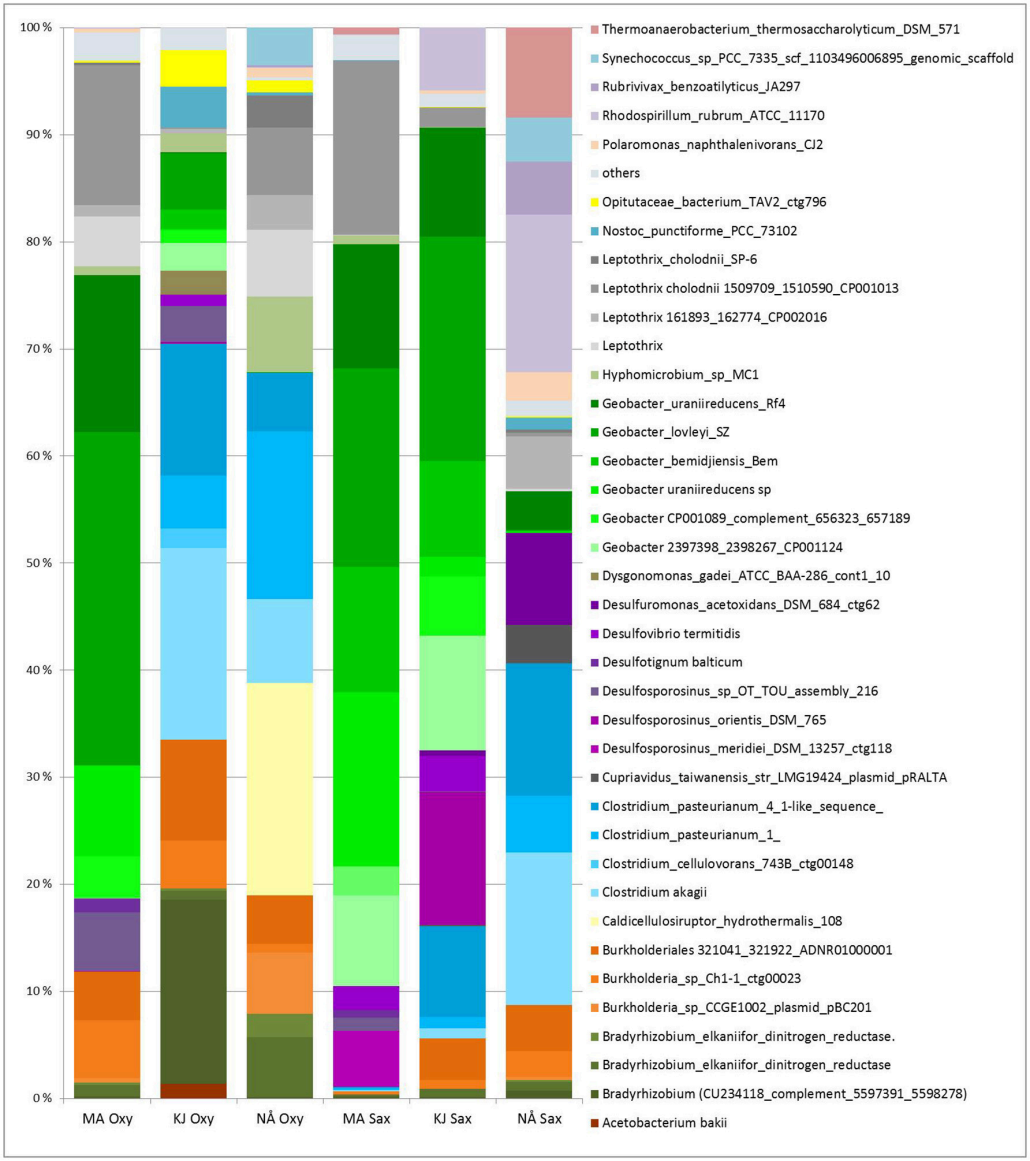
Average relative abundances (four biological replicates) of PNFB in the endospheres of *Oxyria digyna* and S*axifraga oppositifolia* across all three regions. Only major PNFB OPUs (relative abundance above 1%) are indicated, OPUs with <1% relative abundance are grouped together (“others”). Plant species: Oxy, *O. digyna*; Sax, *S. oppositifolia*. Regions: MA, Mayrhofen; KJ, Kilpisjärvi; NÅ, Ny-Ålesund.

Compartment (in particular the endosphere-soil division), followed by region and sampling site, were the major determinants of the plant-associated PNFBs, with no strong host plant specificity. However, we detected small but significant impacts of host plant species on the endosphere PNFB communities (Table 1), and trends in the affiliation of several OPUs with particular plant species were visible. Thus, OPUs representing *Bradyrhizobium* and *Burkholderia* were consistently present in relatively higher abundances in the *O. digyna* endosphere samples than in the *S. oppositifolia* ones (Figure 6). In contrast, the *S. oppositifolia* endosphere communities were more enriched with *Geobacter* related OPUs, in the MA and KJ regions. Furthermore, the latter communities harbored relatively more OPUs related to *Desulfosporosinus* (*Clostridiales*) in KJ and *Clostridium* and *Desulfuromonas* related OPUs in NÅ, in comparison to *O. digyna* (Figure 6). Several of these OPUs were important determinants in shaping the local host plant specific communities (Supplemental Table S1).

## Discussion

Using the *nifH* gene as a proxy, we analyzed the diversity and community composition of PNFB in two pioneer plant species growing in low-nutrient soils in three arcto-alpine climate regions. Based on our previous work, we were aware of the presence of many PNFB taxa representing Firmicutes and δ-proteobacteria, that are routinely missed by many *nifH*-targeting primer combinations (Gaby and Buckley, 2012), in the bacterial communities associated with *O. digyna* and *S. oppositifolia* (Nissinen et al., 2012; Kumar et al., 2017). Thus, we optimized a nested PCR approach with primers 19f/nifH3 (1st round, Yeager et al., 2004) and nifH1f/ nifH2r+nifhH2 (Zehr and McReynolds, 1989; Izquierdo and Nüsslein, 2006). The amplification system was chosen, as it reportedly yields the highest phylogenetic coverage of diazotrophic bacterial groups (Gaby and Buckley, 2012).

Overall, we detected high diversities of PNFB across all samples. Our libraries had good phylogenetic coverage, with the collective PNFB OPUs representing seven phyla (Figure 2), including representatives from *nifH* clusters I, II, and III. This indicates that the selected PCR approach enabled maximal coverage from all the *nifH* phylogenetic groupings (Gaby and Buckley, 2012).

In particular, the PNFB communities in our soils were dominated by *nifH* gene types from the α*-*, β*-*, γ-, and δ-*Proteobacteria* and the *Clostridiales*. The δ-proteobacterial genus *Geobacter* and the β*-*proteobacterial genera *Burkholderia* and *Leptothrix* constituted the most abundant PNFB taxa in the soil communities across all regions. The phylogenetic composition of the PNFB in our data set differs from those reported for high-organic-matter tundra soils from Arctic or alpine sites, in which aerobic proteobacterial diazotrophs dominated (Deslippe and Egger, 2006; Tai et al., 2013).

The endosphere communities in our study were characterized by high relative abundances of *nifH* sequences from the genera *Geobacter, Clostridium, Burkholderia*, and *Bradyrhizobium* (Figures 5A,B). Of these *nifH* sequences clustering with *Geobacter* and *Clostridium* have been discovered in anoxic soils, sediments and wetland rice roots (Rösch et al., 2002; Yeager et al., 2004; You et al., 2005; Wu et al., 2009; Dang et al., 2013). Further, key PNFB taxa in our study, including *G. uraniireducens* Rf4, *G. lovleyi* SZ, and *Optitutaceae* bacterium TAV2, were recently found to be significantly enriched in communities below the water table in thawing permafrost soils in the Canadian Arctic (Penton et al., 2016). Prevalence of anaerobic taxa, also in the plant endosphere communities could be explained by conditions enabling nitrogen fixation under anoxia in most of the soils. Supporting this contention, we observed visible water logging in soils in most of the sampling sites at the time of sampling. Further, soil humidity levels in KJ sampling sites are typically above 36% at early growing season (Kumar, personal observation). We sampled all sites early in the growing season, when the target plants were flowering, which occurred relatively shortly after snowmelt. Both *O. digyna* and *S. oppositifolia* are known to be able to tolerate waterlogging in soil, and they are common snow patch species in low-arctic and alpine soils. It is, thus, likely that the respective plant roots were in the anoxic soil zone, early in the growing season.

Interestingly, *Clostridium* has been reported as an active nitrogen fixer in rice roots in flooded paddy fields (Minamisawa et al., 2004), consistent with our observations. In our study, *Clostridium* was mainly restricted to the endosphere in NÅ and KJ. This is in agreement with our previous analysis of the total bacterial communities of the same plants (based on the16S ribosomal RNA [rRNA] gene) (Kumar et al., 2017), where we observed *Clostridium* spp. to be restricted to the respective endospheres, with increasing relative abundances with increasing latitude in both plant species. Interestingly, 16S rRNA gene sequences of *Clostridium* spp. have been observed in seeds of *O. digyna* (Nissinen, unpublished) and rice (Hardoim et al., 2012), indicating possible vertical transmission.

Confirming our hypothesis, we observed a clear effect of region on the PNFB community structures. Interestingly, PNFB allocated to several *Geobacter* species were present in higher relative abundances in the MA (2,400 m a.s.l.) derived *nifH* sequence data sets. This is in line with reports showing that this taxon is abundant in high mountain ranges, where it may be active under both oxic and anoxic conditions (Duc et al., 2009). Evidence for the presence of PNFB *Geobacter* types has also been found in *nifH* defined communities from switchgrass roots in oxic temperate-climate prairie ecosystems (Bahulikar et al., 2014). This indicates adjustment of this organism to temperate and environmental conditions, including plasticity to function under both aerobic and anaerobic conditions. Curiously, *nifH* sequences related to several *Geobacter* species also dominated the endosphere communities in the MA region, while they were largely replaced by those from *Clostridium* and other PNBF taxa in the Arctic (Figure 6). As all the main PNFB OPUs were detected in all regions, this strong regionality cannot be explained by an absence of *Geobacter* in the Arctic or of *Clostridium* in the Alps. Rather, differential selection by plants or different conditions in the endorhiza in different regions could be the driver.

One of the main differences between arctic and alpine biomes is the better drainage of alpine soils compared to Arctic ones (Körner, 2003). The NÅ sites used in this study are underlaid by permafrost, and the KJ sites are characterized by patchy permafrost, with snow often melting away as late as mid-July, leading to water-logged conditions, at least early in the growing season. The alpine MA sites, on the other hand, were not water-logged at the time of the sampling. This observation could explain the differential abundance of several PNFB taxa in the different regions, also in the endosphere.

Corroborating the 16S rRNA gene based analyses reported earlier, endophytic bacterial taxa that were previously identified as key taxa in the total endosphere bacterial communities in these same plants, e.g., *Bradyrhizobium, Burkholderiales, Comamonadaceae*, and *Clostridia* (Kumar et al., 2017), were found to also make part of the PNFB endosphere communities, indicating an important role for the nitrogen fixing bacteria for these plants common in pioneer communities.

Partially confirming our hypothesis, we detected a significant, but small effect of host plant species (*O. digyna* and *S. oppositifolia)* on the endophytic PNFB communities across regions (Table 1). The difference between PNFB communities of the two plant species was smaller than the previously reported effect on total bacterial community structures (Kumar et al., 2017). This is likely due to strong regionality in the PNFB community structures. However, the PNFB genera that showed plant species specific accumulation in the individual regions (for example *Burkholderia* in *O. digyna* and *Clostridium* in *S. oppositifolia*) were also identified as showing host plant specificity in 16s rRNA based analyses of total bacterial communities of these same plants (Kumar et al., 2017).

We provided in this paper a comprehensive overview of PNFB associated with two pioneer plant species in three arcto-alpine climate zones. Our collective data show that the PNFB communities associated with these plants in three different cold-climate regions are subjected to selective processes that operate at different scales, i.e., that of compartment and that of region and climate. On the basis of the high relative abundances of anaerobic nitrogen fixers in these communities, we hypothesize that the local conditions allowing nitrogen fixation at the plants might have been prevalently anoxic in the two arctic regions, whereas those in the alpine region may have fluctuated between prevalently oxic or anoxic conditions. These PNFB communities, representing pioneer communities that are likely to become more common in near future due to changing climate, differ clearly from plant associated PNFB communities described in different climates and in high organic matter tundra soils.

## Nucleotide sequence data

Nucleotide sequence data have been submitted to the ENA database with accession number PRJEB20618.

## Author contributions

Study was conceptualized and designed by RN, MK, and JvE. Field work was performed by MK and RN. Sample processing was done by MK and RN. Library preparation for sequence analysis was done by MK, and data analysis was done by MK and RN. Manuscript draft was prepared by MK, RN, and JvE, and revisions were done by MK, RN, and JvE. Final version for the submission was prepared by RN and JvE.

### Conflict of interest statement

The authors declare that the research was conducted in the absence of any commercial or financial relationships that could be construed as a potential conflict of interest.
